# Myelin damage and repair in pathologic CNS: challenges and prospects

**DOI:** 10.3389/fnmol.2015.00035

**Published:** 2015-07-27

**Authors:** Arsalan Alizadeh, Scott M. Dyck, Soheila Karimi-Abdolrezaee

**Affiliations:** Regenerative Medicine Program, Department of Physiology and Pathophysiology, Spinal Cord Research Centre, Faculty of Health Sciences, College of Medicine, University of Manitoba, WinnipegMB, Canada

**Keywords:** demyelination, spinal cord injury, cell therapy, oligodendrocytes, remyelination, neural stem cells, oligodendrocyte precursor cells, astrocytes

## Abstract

Injury to the central nervous system (CNS) results in oligodendrocyte cell death and progressive demyelination. Demyelinated axons undergo considerable physiological changes and molecular reorganizations that collectively result in axonal dysfunction, degeneration and loss of sensory and motor functions. Endogenous adult oligodendrocyte precursor cells and neural stem/progenitor cells contribute to the replacement of oligodendrocytes, however, the extent and quality of endogenous remyelination is suboptimal. Emerging evidence indicates that optimal remyelination is restricted by multiple factors including (i) low levels of factors that promote oligodendrogenesis; (ii) cell death among newly generated oligodendrocytes, (iii) inhibitory factors in the post-injury milieu that impede remyelination, and (iv) deficient expression of key growth factors essential for proper re-construction of a highly organized myelin sheath. Considering these challenges, over the past several years, a number of cell-based strategies have been developed to optimize remyelination therapeutically. Outcomes of these basic and preclinical discoveries are promising and signify the importance of remyelination as a mechanism for improving functions in CNS injuries. In this review, we provide an overview on: (1) the precise organization of myelinated axons and the reciprocal axo-myelin interactions that warrant properly balanced physiological activities within the CNS; (2) underlying cause of demyelination and the structural and functional consequences of demyelination in axons following injury and disease; (3) the endogenous mechanisms of oligodendrocyte replacement; (4) the modulatory role of reactive astrocytes and inflammatory cells in remyelination; and (5) the current status of cell-based therapies for promoting remyelination. Careful elucidation of the cellular and molecular mechanisms of demyelination in the pathologic CNS is a key to better understanding the impact of remyelination for CNS repair.

## Introduction

Myelin is a cholesterol rich extension of oligodendrocytes and Schwann cells (SCs) plasma membrane, which serves as a specialized insulation sheath for axons in the nervous system. Myelin facilitates axon signal conduction through enabling “saltatory conduction” (see review by Miron and Franklin, 2014). However, the importance of myelin in the central nervous system (CNS) is beyond its role in rapid signal conduction along axons as its disturbance also cause other severe functional and neurobehavioral disabilities (as reviewed by Love, 2006). Myelin is important for axon maintenance and function (Nave and Trapp, 2008). Perturbations of myelin structure and function or “demyelination” is associated with a long list of CNS pathologies from congenital and autoimmune disorders to metabolic disturbances (Love, 2006). Progressive demyelination also results in axonal degeneration due to the disruption of axo-oligodendrocyte signaling. A healthy cross talk between axons and oligodendrocytes is required to maintain proper metabolic function of axons, trophic support, cytoskeletal arrangement, ion channel organization, and axonal transport (Edgar et al., 2004; Devaux and Scherer, 2005; Kassmann and Nave, 2008; Nave and Trapp, 2008; Bruce et al., 2010; Nave, 2010; Fünfschilling et al., 2012). Axons become dependent on myelinating glia as myelin appears during the development. This concept was demonstrated in PLP/DM20 deficient mice where the absence of these essential myelin proteolipids resulted in axonal swellings only in myelinated axons with no evidence of axonal pathology in normal unmyelinated fibers (Griffiths et al., 1998). Moreover; mice lacking *Cnp1*, which encodes 2′,3′-cyclic nucleotide phosphodiesterase in oligodendrocytes, show no structural abnormality in myelin but develop axonal swelling and degeneration (Lappe-Siefke et al., 2003). These studies suggest that myelinated axons receive signals from oligodendrocytes that trigger their dependency to myelin. Interestingly, such a dependency have not yet been observe in non-myelinated axons (Griffiths et al., 1998; Lappe-Siefke et al., 2003). Survival of oligodendrocytes is also dependent on axons. Following injury, oligodendrocytes distal to the site of an axonal injury degenerate due to lack of trophic support from the injured axon (Lappe-Siefke et al., 2003). Considering the reciprocal axo-oligodendrocytes signaling, replacement of oligodendrocytes and renewal of myelin sheath around surviving demyelinated axons following injury is a vital repair strategy for CNS regeneration and functional recovery.

Oligodendrocyte precursor cells (OPCs) and neural stem/progenitor cells (NPCs) are two endogenous cell populations, capable of replacing lost oligodendrocytes and remyelinating spared axons following injury (Beattie et al., 1997; Eftekharpour et al., 2008; Meletis et al., 2008; Barnabe-Heider et al., 2010). Despite the spontaneous response and activation of both OPCs and NPCs upon injury, adequacy, and quality of remyelination is challenged due to multiple factors including modifications in the extracellular matrix, astrogliosis, and downregulation of essential trophic and growth factors (Karimi-Abdolrezaee et al., 2006; Meletis et al., 2008; Barnabe-Heider et al., 2010; Karimi-Abdolrezaee et al., 2012; Lau et al., 2012; Gauthier et al., 2013; Lukovic et al., 2015). These injury-induced events either limit oligodendrocyte differentiation or impede the process of axonal ensheathment and remyelination. Over the last decade, cellular and pharmacological repair strategies have been developed to induce remyelination by recruiting endogenous precursor cells or through stem cell therapies (Karimi-Abdolrezaee et al., 2006; Eftekharpour et al., 2007, 2008; Joubert et al., 2010; Kotter et al., 2011; Karimi-Abdolrezaee and Eftekharpour, 2012; Rodgers et al., 2013; Plemel et al., 2014). In this review, (1) we will provide an overview on the precise molecular and ion channel organization of myelinated axons and the reciprocal axo-myelin interactions that warrant properly balanced physiological activities within the CNS, (2) we will dissect the underlying cause of demyelination and the structural and functional consequences of demyelination in axons by focusing on spinal cord injury (SCI) and multiple sclerosis (MS) models, (3) we will discuss the role of activated glia in demyelination and remyelination following demyelination, and (4) we will review the current status of cell-based therapeutic interventions that are designed to promote oligodendrocyte differentiation and facilitate remyelination. Understanding the functional ramification of demyelination and remyelination and the cellular and molecular basis of these events will aid in developing targeted therapies to more effectively promote myelin repair and prevent disease progression in demyelinating conditions.

## Normal Molecular Organization of Myelin and Nodes of Ranvier

Myelin is a modified plasma membrane of oligodendrocytes in the CNS, which enwraps a segment of axon in a spiral fashion (Barres et al., 1993). Myelination affects function and molecular organization of axons allowing faster signal propagation with reduced energy consumption (Frankenhaeuser and Schneider, 1951; Bishop and Levick, 1956; Homma et al., 1983; Saab et al., 2013; Stiefel et al., 2013). Several proteins in myelin have been identified to play essential roles in axonal maintenance and function. Proteolipid protein (PLP) and its spliced derivative, DM20, are essential for proper axonal function (Griffiths et al., 1998; Lappe-Siefke et al., 2003). Loss of either PLP or DM20 will affect myelin periodicity and cause axonal swelling (Griffiths et al., 1998; Lappe-Siefke et al., 2003). Swollen axons will gradually become dysfunctional and degenerate causing functional deficits at later stages (Griffiths et al., 1998). Myelin basic protein (MBP) is another structural protein that plays a vital role in myelin compaction and thickening in the CNS (Condorelli et al., 2003; Eftekharpour et al., 2007). *Shiverer* mice that lack MBP demonstrate dysmyelinated axons associated with axonal dysfunction and motor impairments (Loers et al., 2004; Sinha et al., 2006). Interestingly, *Shiverer* mice do not develop axonal swelling and show minimal axonal degeneration compared to PLP/DM20 deficient mice even up to 2–3 months following birth (Griffiths et al., 1998; Loers et al., 2004). Myelin associated glycoprotein (MAG) is essential for the initiation of myelination (Biffiger et al., 2000). Mice with double knockout of MAG and Fyn (a downstream signaling molecule in MAG/Fyn pathway) demonstrate severe optic nerve hypomyelination despite the unaffected presence of oligodendrocytes (Biffiger et al., 2000). MAG is also known to be essential for survival and integrity of myelinated axons (Yin et al., 1998; Pan et al., 2005; Nguyen et al., 2009), however, such a role has not been established for Fyn (Biffiger et al., 2000). CNPase (2,3-cyclic nucleotide 3-phosphodiesterase) is an enzyme that is synthesized in myelinating mature oligodendrocytes and can be found in non-compact regions of the myelin sheath (Nagy et al., 1997). Lack of CNPase has not been shown to affect myelination but myelinated axons will eventually become swollen and degenerate (Lappe-Siefke et al., 2003; Rocco et al., 2004). This evidence demonstrates the importance of the various myelin compartments/proteins for the proper functioning of axons and oligodendrocytes. However, further investigations are required to elucidate the role of each myelin protein in this complex relationship.

**FIGURE 1:**
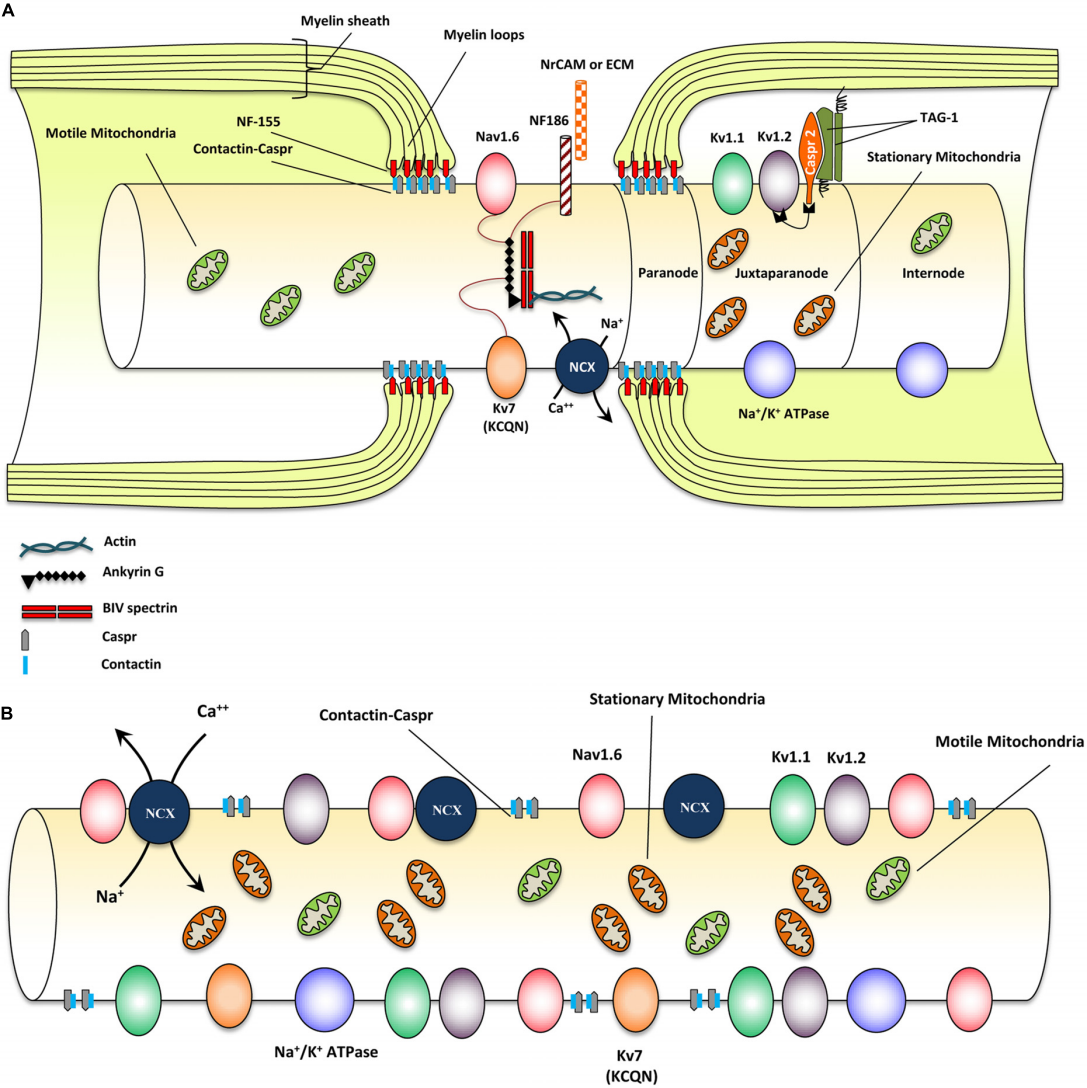
**Structural and molecular organization of myelinated axons in normal and demyelinating conditions. (A)** Schematic diagram shows structure and molecular configuration of a myelinated axon at the node of Ranvier, paranodal and juxtaparanodal regions. Nav 1.6 and Kv7 (KCQN) are located in the nodal region and are essential for formation and propagation of action potential. Na^+^/Ca^2+^ exchanger (NCX) is also located in nodal area and exchanges intracellular sodium with extracellular Ca^2+^ in an ATP dependent manner. Ion channels are precisely localized to specific domains of axons through their contact with adhesion molecules such as neurofascin (NF)-186. These adhesion molecules aid in stabilizing ion channels by connecting them with extracellular matrix (ECM) and glial cell processes surrounding the nodal region. Paranodal junctions are the region where myelin loops are tethered to axonal membranes. Contactin and contactin associated protein (Caspr) play key roles in formation of paranodal loops through their interaction with neurofascin (NF)-155 and other adhesion molecules from myelinating glia. Juxtaparanode contains voltage gated Kv^+^ channels (Kv1.1 and 1.2) that are essential for restoring resting membrane potential. Kv channels allow for potassium to exit the axons quickly following depolarization. Caspr2/TAG-1 adhesion complex stabilizes these Kv1.1 and Kv1.2 channels in axonal membrane. Stationary mitochondria (brown) are mainly located in juxtaparanodal and internodal regions where Na/K ATPases are abundant to provide energy for ion homeostasis. There is another mitochondrial population called motile mitochondria (green) which can translocate in both retrograde and anterograde directions along the axon. These mitochondria are being produced in the cell body and can stop in stationary sites. They are important for the turnover and redistribution of mitochondria along the axons and during changes in energy demand. **(B)** Following demyelination, due to the disruption of paranodal myelin loops, all ion channels, pumps and exchangers become dispersed along the axon and sodium influx increases through Nav1.6 channels. Expression of Nav1.6, Kv1.1, and Kv1.2 channels increases significantly following demyelination. Sodium overload causes axonal calcium to reach toxic levels as it is being exchanged with sodium through NCXs by an energy dependent process. Following demyelination, speed of mitochondrial transportation and size of stationary mitochondria significantly increase to compensate for the increased energy demand. Despite robust increase in mitochondrial content, demyelinated axons are unable to maintain a balance between their energy production and expenditure that results in axonal degeneration eventually.

Myelinated axons show a high degree of structural organization. A myelinated axon can be separated into distinct domains including node of Ranvier, paranode, juxtaparanode, and internode (Eftekharpour et al., 2008; Ohno et al., 2014; Plemel et al., 2014) (**Figure 1A**). Node of Ranvier is the gap between two adjacent myelin sheaths and contains high concentrations of voltage-dependent Na^+^ channels on the axonal membrane (Amor et al., 2014). Electrical impulse cannot flow through the high resistance myelin sheath, but instead flows through the node of Ranvier and depolarizes the axonal membrane at each node resulting in saltatory conduction (Ohno et al., 2011).

In myelinated axons, node of Ranvier was characterized by the localization of voltage-gated sodium (Na_v_) and KCNQ K^+^ channels (Chiu and Ritchie, 1980; Rasband et al., 1998). Node of Ranvier also contains a collection of adhesion molecules, adaptor proteins, and cytoskeletal structures including, βIV-spectrin, ankyrin G, neuron-glia-related cell adhesion molecule (NrCAM) and a 186 kDa isoform of neurofascin (NF186) (Davis et al., 1996; Salzer, 2003; Amor et al., 2014) (**Figure 1A**). Among these molecules, βIV-spectrin and ankyrin G play a major role in stabilizing the Na_v_ channels at nodal region (Lai and Jan, 2006). During the development of axons, Na_v_1.2 channels are initially expressed along pre-myelinated axons with the capability to generate an action potential (Caldwell et al., 2000; Rasband and Shrager, 2000). As myelination ensues, Na_v_ 1.6 channels begin to cluster at mature nodes of Ranvier (Boiko et al., 2001; Kaplan et al., 2001). Na_v_1.2 and Na_v_1.6 channels are both rapidly activating and inactivating channels but Na_v_1.6 is known to produce a larger persistent current (Caldwell et al., 2000; Rush et al., 2005). Glial cells play an essential role in the formation of normal nodes of Ranvier with their typical nodal Na_v_ and paranodal K_v_ channel distribution. As it has been reviewed by Schafer and Rasband (2006), there are similarities in the contribution of glial cells in node formation between the CNS and PNS. In both systems glial cell adhesion molecules (CAMs) in close association with axonal CAMs and cytoskeletal domain, form a structural framework that clusters ion channels with specific formation in nodal and paranodal areas (Schafer and Rasband, 2006) (see **Figure 1A**).

Paranode is the adjacent segment to the node of Ranvier where myelin loops provide an anchor by tethering the myelin to the axonal membrane (Poliak et al., 1999). Evidence has established a determining role for paranodal axo-oligodendrocyte junction in precise localization of ion channels into specialized domains of myelinated axons (Barres et al., 1992a,b; Davis et al., 1996; Kiernan et al., 1996; Kaplan et al., 1997). Paranodal junctions are critical in preventing lateral diffusion of ion channels along the axons to ensure proper segregation of Na^+^ and K^+^ channels at discrete domains on axonal membrane (Gard et al., 1995; Peles and Salzer, 2000). The paranodal region was characterized by the presence of Contactin and Contactin-Associated Protein (Caspr) that form a complex in the “septate-like junctions” between myelin loops and axolemma (Einheber et al., 1997). Caspr is critical for the establishment of axo-glia junction in myelinated fibers through its interactions with contactin and NF-155 (Lyons and Talbot, 2008). Caspr deficiency results in disruption of the paranodal region and aberrant distribution of ion channels along the axons (Kiernan et al., 1996) (see **Figure 1A**).

The juxtaparanode contains delayed-rectifier *voltage-gated* Kv^+^ channels and Na^+^/K^+^ ATPase channels that allow for rapid exchange of axoplasmic Na^+^ for extracellular K^+^ and restoration of the resting membrane potential (Poliak et al., 2003; Traka et al., 2003). As ion channel clustering evolves, shaker type Kv1.1 and Kv1.2 channels begin to localize in juxtaparanodal region of the myelinated axons (Wang et al., 1993; Rasband et al., 1998). These channels are associated with the Caspr2/TAG-1 adhesion complex (Poliak et al., 1999, 2003; Traka et al., 2002, 2003; Horresh et al., 2008) (**Figure 1A**). Upon receiving of an action potential, Na_v_ channels open, allowing an influx of Na^+^ into the axon causing depolarization. After each depolarization, Na^+^/K^+^ ATPase pumps, located at the juxtaparanodal and internodal regions, exchange axonal Na^+^ for extracellular K^+^ (Meta et al., 1991). This process is an energy dependent mechanism and is essential for rapid and repetitive axonal firing. Similarities exist between the molecular organization of nodes and the axon initial segment; however, while myelin is crucial for the proper molecular organization of nodes, the axon initial segment appears to be intrinsically organized by the neuron (Dzhashiashvili et al., 2007; Yang et al., 2007). Evidence from our group and others have shown that demyelination due to injury and disease results in disruption of the precise nodal organization causing axonal dysfunction (Davis et al., 1996; Kaplan et al., 1997; Nashmi et al., 2000; Karimi-Abdolrezaee et al., 2004; Eftekharpour et al., 2005, 2007; Sinha et al., 2006). Additionally, myelination provides extrinsic trophic signals, which influence the normal maturation, maintenance, and long-term survival of axons (White et al., 2009; Mar and Noetzel, 2010; Castelvetri et al., 2011; Lassmann et al., 2012; Mekhail et al., 2012; Teixeira et al., 2014). Structural and functional importance of nodal organization will be discussed in subsequent sections.

## Demyelination and Its Pathophysiological Consequences

Demyelination is damage or loss of the myelin sheath around axons. It is mainly a consequence of oligodendroglia cell death that can occur through multiple mechanisms depending on the type of disease or injury, including genetic defects, infectious agents, autoimmune reactions, trauma, and some by unknown mechanisms (Zimmerman, 1956; Popescu and Lucchinetti, 2012; Kutzelnigg and Lassmann, 2014). Several genetic disorders exist that can cause defects in myelin through improper myelination and myelin maintenance or progressive demyelination over time. Charcot-Marie-Tooth disease (CMT), Alexander disease, and Krabbe disease are examples of the many known genetic diseases characterized by axonal demyelination or dysmyelination (Ida et al., 1990; Satoh et al., 1997; Rocco et al., 2004; da Silva Pereira et al., 2013; Perveen et al., 2015).

Multiple Sclerosis is a classic example of autoimmune demyelination in the CNS (Bitsch et al., 2000; Kuhlmann et al., 2008). The early stages of MS involve relapsing-remitting where patient experience demyelination associated with loss of function (i.e., vision and gait), which is usually regained following remyelination. In the progressive stages of MS, irreversible functional deficit occurs which has been associated with progressive loss of axons and neurons (Kurtzke et al., 1977; Flachenecker and Hartung, 1996). Degeneration of chronically demyelinated axons is now considered to be a major contributor to the permanent neurological disability that MS patients eventually endure (Saito et al., 1990; Bjartmar and Trapp, 2001, 2003; Stys, 2004; Su et al., 2009; Dziedzic et al., 2010; Cambron et al., 2012).

Demyelination can also occur through traumatic injury. In the chronically injured spinal cord, there is varying degree of demyelination and dysmyelination in the subpial rim surrounding the lesion site (Nashmi and Fehlings, 2001). Following SCI, some axons and oligodendrocytes are initially lost through necrosis due to mechanical injury. As injury evolves, progressive loss of oligodendrocytes occurs through apoptosis and autophagy that results in demyelination of injured spared axons (Abe et al., 1999; Casha et al., 2001; McTigue and Tripathi, 2008; Kanno et al., 2009; Plemel et al., 2014). Remyelination occurs spontaneously by both OPCs and NPCs following injury even in the chronically injured spinal cord (Beattie et al., 1997; Salgado-Ceballos et al., 1998; Hesp et al., 2015). However, this remyelination attempt is often limited and inadequate due to changes to the post-injury environment (Barnabe-Heider et al., 2010; Karimi-Abdolrezaee et al., 2010, 2012; Xing et al., 2014; Hesp et al., 2015). Therapeutic strategies aimed at promoting remyelination have demonstrated the potential to promote axonal sparing and limit progressive axonal dieback in chronic SCI (Karimi-Abdolrezaee et al., 2010).

Animal models of demyelinating disease such as MS provide invaluable tools to study myelin–axon interactions and understand the pathological effects of demyelination on axonal integrity and function. Here, we will primarily focus on the effects of demyelination on axons in models of MS and SCI, however, many of the details provided here also correlate with other findings in the literature in other demyelinating conditions.

### Changes to Ion Channels Following Demyelination

Loss of myelin sheath causes aberrant distribution of ion channels, where Na_v_ channels diffuse away from the nodes and redistribute across the axonal surface (Waxman et al., 2004). Additionally, there appears to be an overall increase in the expression of Na_v_ channels in chronically demyelinated axons (Bostock and Sears, 1978; Foster et al., 1980; Waxman et al., 2004). Following experimental autoimmune encephalomyelitis (EAE), Na_v_1.2, and Na_v_1.6 channels are up-regulated in demyelinated axons (Craner et al., 2003, 2004a) (see **Figure 1B**). Sodium channel redistribution causes an overall increase in Na^+^ influx during impulse conduction and increased demand for ATP during repolarization (Waxman et al., 2004). Furthermore, increased sodium influx has been associated with axonal degeneration through a Ca^2+^-mediated effect by causing the reversal of the Na^+^/Ca^2+^ exchanger (NCX) (Stys et al., 1992; **Figure 1**). Inhibition of sodium channels and NCX has been shown to prevent axonal degeneration (Rosenberg et al., 1999; Bechtold et al., 2004; Hains et al., 2004). Increased axonal Ca^2+^ can activate proteolytic enzymes and eventually lead to degeneration of chronically demyelinated axons (Stys et al., 1992). Ca^2+^ influx is not normally toxic in axons, however, increased energy demand and thus lack of ATP in axons following demyelination causes failure in the energy-dependent Ca^2+^ buffering system to efficiently remove excess Ca^2+^. This results in a rise in the Ca^2+^ concentration to toxic levels (Stys and Lopachin, 1998; Trapp and Stys, 2009; Plemel et al., 2014). Lack of ATP in chronically demyelinated axons is thought to render axons vulnerable to cellular death over time (Stys, 1998).

Axonal degeneration caused by sodium influx is thought to be mainly mediated through Na_v_1.6 channels. Na_v_1.6 channels produce a persistent current, which is much larger than that of Na_v_1.2 (Smith et al., 1998). Na_v_1.6 has been shown to be co-localized with NCX following demyelination of axons in EAE model (Craner et al., 2004a) and in postmortem cervical spinal cord and optic nerve tissue of acute MS patients (Craner et al., 2004b). Na_v_1.6 is co-localized in 60% of axons, which express β-amyloid precursor protein (APP), a marker for axonal injury, whereas it is only expressed in 20% of axons, which are β-APP negative (Craner et al., 2004b). Importantly, in chronic lesions of MS, Na_v_1.6 is expressed in patches in only a third of axons (Black et al., 2007). Evidence shows that demyelinated axons are more susceptible to axonal injury than dysmyelinated axons, which may be explained by the altered expression of Na_v_1.6. Dysmyelination in MBP^-/-^ *Shiverer* mice have altered expression of Na_v_ channels in their axons with Na_v_1.2 being retained into adulthood and Na_v_1.6 not being expressed (Boiko et al., 2001). Similarly, PLP^-/-^ mice also show a loss of Na_v_1.6 channel clustering and an increased expression of Na_v_1.2 (Rasband et al., 2003). Because Na_v_1.2 channels are expressed in pre-myelinated axons and can produce signals, it is believed that newly produced Na_v_1.2 channels support conduction in demyelinated axons (Craner et al., 2004b).

Juxtaparanodal voltage-gated Kv^+^ channels are also influenced by demyelination after SCI or genetic dysmyelination (Nashmi et al., 2000; Karimi-Abdolrezaee et al., 2004; Eftekharpour et al., 2005, 2007). We have shown that spinal cord axons in dysmyelinated *Shiverer* mice exhibit a dispersed distribution of Kv^+^ channel subunits Kv1.1 and Kv1.2 associated with the loss of the characteristics of juxtaparanodal and paranodal structures (Eftekharpour et al., 2007) (**Figure 1B**). Our investigations on dysmyelinated *Shiverer* mice and Long Evans Shaker (LES) rats elucidated the role of K^+^ channels in axonal function (Eftekharpour et al., 2005; Sinha et al., 2006). These studies revealed aberrant localization and increased expression of both Kv1.1 and Kv1.2 channel subunits along axolemma of dysmyelinated axons while these channels were confined to juxtaparanodal regions of the wild-type axons. Using sucrose gap recording of spinal cord monophasic compound action potentials (CAP), we demonstrated that *Shiverer* spinal cord axons have significantly lower CAP amplitude and area compared to wild-type counterparts (Sinha et al., 2006). Kv^+^ channel blockage by specific (DTX-I, DTX-K) and non-specific (4-AP) blockers improved axonal conductance; however, this effect was shown to be more dependent on a combination of subunits as a specific blocker of Kv1.1 failed to improve axonal conduction significantly (Sinha et al., 2006). Interestingly, Kv^+^ channels are also important for remyelination (Bacia et al., 2004). Administration 4-aminopyridine (4-AP), a broad-spectrum K^+^ channel antagonist that blocks fast K^+^ channels, to a mouse model of cuprizone-induced demyelination, resulted in impaired oligodendrocyte regeneration and remyelination (Bacia et al., 2004). These studies collectively demonstrate the pivotal role of axo-myelin interactions in ion channels distributions and functions and more importantly on axonal physiology.

### Effect of Demyelination on Axonal Transport and Metabolism

Axonal transport shuttles critical cell body-derived components back and forth between the soma and axon and across synapses in neurons (Millecamps and Julien, 2013). Dysfunction of axonal transport causes neuronal homeostasis imbalance and as a result makes axons more susceptible to axonal degeneration. Axonal transport disturbances are thought to precede the initiation of neurodegeneration in diseases including, hereditary spastic paraplegias (HSPs), AD and Huntington’s disease (Gunawardena et al., 2003; Ebbing et al., 2008; Millecamps and Julien, 2013). Accumulation of APP is known as; an early marker of injury in MS patients and is believed to accumulate due to lack of axonal transport following injury (Ferguson et al., 1997; Smith et al., 2003).

Oligodendrocytes and their myelin sheath are critical in regulating slow and fast anterograde transport rates (Kirkpatrick et al., 2001; Edgar et al., 2004). Reduced fast axonal transport can cause degeneration in distal parts of the axons as observed in X-linked spastic paraplegia type 2, which is caused by a mutation of the PLP1 gene, a major protein of the myelin sheath. Absence of PLP causes swelling of axons and deficits in retrograde and anterograde transport (Griffiths et al., 1998; Edgar et al., 2004). In both humans and mice, absence of PLP causes selective axonal degeneration of long tracts including the fasciculus gracilis and distal corticospinal tracts (Garbem et al., 2002). Conversely, *Shiverer* mice, lacking MBP, demonstrate a significant increase in slow axonal transport associated with increased density and instability of microtubules in axons (Kirkpatrick et al., 2001).

Demyelination-induced defects in axonal transport has been also detected in MS models (Lin et al., 2014; Sorbara et al., 2014). Studies in an EAE model of optic neuritis in rats suggest that the extent of disruption in axonal transport appear to be correlated with the severity of inflammation, demyelination, and axonal injury (Lin et al., 2014). Live imaging of individual axonal organelles in the spinal cord of mice with acute EAE revealed that the anterograde and retrograde transport of mitochondria and peroxisomes were markedly reduced in spinal axons, which passed through the lesion (Sorbara et al., 2014). In chronic model of MS, when axonal transport is restricted, there is reduced density in distal organelles which can ultimately lead to “starvation” of distal axonal arbors and axonal degeneration (Sorbara et al., 2014). Transport deficits were shown to occur prior to any marked alteration of microtubule tracks (Sorbara et al., 2014). Dysfunction of axonal transport recovers within days following insult. However, in chronic MS lesions, transport deficits were apparent resulting in lack of distal organelle supply (Sorbara et al., 2014). Reduced axonal transport has been attributed to reactive oxygen and nitrogen species released by immune cells, which alters the attachment of motor/cargo complexes to microtubules (Sorbara et al., 2014). In chronic MS models, anterograde transport from the soma to the synapses appears to be considerably affected resulting in reduced organelle transport from cell body to the axonal terminal at synapses (Sorbara et al., 2014). Hence, interventions to restore transport tracks and axonal transport seem to be vital strategies to slow down the progression of axonal degeneration and reverse these degenerative effects (d’Ydewalle et al., 2011; Denton et al., 2014; Sorbara et al., 2014).

The majority of white matter tract axons were entirely enwrapped by myelin; therefore, it is likely that axons cannot obtain proper nutrient support from their external environment on their own and require metabolic support from glial cells (Saab et al., 2013). It is currently unknown whether glucose transporters are present at the node of Ranvier (Saab et al., 2013). In axons, the highest energy requirement occurs at the Na^+^/K^+^ ATPase pump, which is located along the internodes (Meta et al., 1991). It is proposed that axons receive their metabolic support mainly through the “pan-glial” network of oligodendrocytes and astrocytes (Saab et al., 2013). Astrocytes were further connected to the blood–brain-barrier and to the nutritive support by brain capillaries (Karimi-Abdolrezaee and Billakanti, 2012). Oligodendrocytes, expressing connexin (Cx) 47, were coupled to astrocytes, expressing Cx30, through gap-junctions (Tress et al., 2012). Double KO mice of Cx47 and Cx30 results in axonal loss, and death in mice (Tress et al., 2012). This evidence suggests that myelination has a dual role in supporting the metabolic activity of neurons by saving energy of axons through saltatory conduction and providing nutrients to neurons.

Ensheathment of axons by oligodendrocytes are shown to drastically diminish the ATP consumption of neurons by reducing the energy required by axons to transmit signals over long distances through saltatory conduction (Barron et al., 2004). However, is it important to take into consideration that the metabolic cost of myelin synthesis and maintenance might be higher than the saved energy (Harris and Attwell, 2012). Nonetheless, myelin does save the amount of energy required in neurons by decreasing the energy expenditure required to maintain its resting membrane potential and to propagate signals (Saab et al., 2013). Following demyelination, there is an overall increased demand for ATP (Andrews et al., 2006). The energy required to maintain the intra-axonal ionic balance and the resting membrane potential by the Na^+^/K^+^ ATPase pump were increased, due to redistribution and an overall increase in the number of Na^+^ channels (Craner et al., 2004a; Black et al., 2007). *Shiverer* mice exhibit a significant change in the density and activity of mitochondria in their axons in comparison to wild type animals (Andrews et al., 2006). Similarly, unmyelinated segments of retinal ganglion cell axons in the lamina cribrosa also show increased metabolic activity in comparison to myelinated segments (Bristow et al., 2002; Barron et al., 2004). Increased metabolism requirements for demyelinated axons may make these axons more susceptible to death through disease mechanisms such as inflammation (Millecamps and Julien, 2013).

Mitochondria are the major source of axonal ATP and play a critical role in apoptosis, reactive oxygen species generation and calcium buffering (Sheng and Cai, 2012). Two separate populations of mitochondria exist in myelinated axons, stationary and motile mitochondria. The majority of mitochondria are located throughout the axons in stationary sites where multiple mitochondria reside (Ohno et al., 2011; Saxton and Hollenbeck, 2012). Stationary mitochondrial are predominantly located in juxtaparanodal/internodal axoplasm, containing multiple morphologies of varying length, whereas those located in the nodes/paranodal axoplasm appear uniformly short or absent altogether (Ohno et al., 2011). Energy consumption in axons is highest at the juxtaparanodal and internodal regions where Na^+^/K^+^ ATPases are abundantly present (Meta et al., 1991). These stationary mitochondria do not translocate and usually vary in length but typically contain the same diameter throughout the population (Ohno et al., 2011). A separate population of relatively small but motile mitochondria also exist which translocate in both anterograde and retrograde directions (Detmer and Chan, 2007). These motile mitochondria are produced in the cell body, and can stop within stationary sites. They are essential for the turnover and redistribution of mitochondria and have been shown to fuse with or bud from stationary mitochondria (Detmer and Chan, 2007; Berman et al., 2009; Sheng and Cai, 2012). The rate of transport and docking of these motile mitochondria can be influenced by axonal metabolic demand, such as increases in axonal firing (Ohno et al., 2011).

Recent evidence has shed some light onto the changes that occur to mitochondria in the acute stages of demyelination. Following demyelination there is an overall increased demand for ATP mainly due to changes in ionic homeostasis (Barron et al., 2004). Moreover, the size of stationary sites and the speed of mitochondrial transport is increased in demyelinated axons (Kiryu-Seo et al., 2010). *In vitro* studies on myelinated rat dorsal root ganglion (DRG) axons showed 2.2-fold increase in the size of stationary mitochondria sites and 47% increase in the velocity of motile mitochondria following demyelination (Kiryu-Seo et al., 2010). This response is shown to be an axonal response to the increased ATP demand of demyelinated axons mediated, at least partially, through activating transcription factor 3 (Kiryu-Seo et al., 2010). Increased volume of mitochondria at these stationary sites are shown to be a protective response by demyelinated axons mediated through syntaphilin, a protein, which tethers mitochondria to microtubules at stationary sites (Ohno et al., 2014). Chronically demyelinated axons exhibit increased expression of syntaphilin (Mahad et al., 2009). Demyelinated axons deficient in syntaphilin degenerate at a significantly greater rate than wild type axons associated with smaller increases in stationary mitochondrial volume indicating the importance of mitochondrial migration to these stationary sites (Ohno et al., 2014). In summary, increasing mitochondrial stationary site size is important in protecting neurons from degeneration following CNS demyelination (Kiryu-Seo et al., 2010; Ohno et al., 2014).

Despite this protective response from axons, mitochondrial function appears to be limited in chronically demyelinated lesions of MS (Sheng and Cai, 2012). Following demyelination, changes to the energy balance in axons and dysfunctions of axonal mitochondria contribute to degeneration of chronically demyelinated axons (Sheng and Cai, 2012). There appears to be an overall decrease in the ability of neurons to produce ATP through their mitochondria (Dutta et al., 2006; Mahad et al., 2009; Campbell et al., 2011). In postmortem MS tissues, there was a decreased expression of mitochondrial electron transport chain genes which was associated with decreased ability of mitochondria to exchange electrons in respiratory chain complex I, III (Dutta et al., 2006), and IV (Mahad et al., 2009). This decrease in respiration was later shown to be mediated through deletion of mitochondrial DNA in axons (Campbell et al., 2011). These data suggest that mitochondria in the chronically demyelinated axons have a reduced ability to produce ATP, which can contribute to the axonal degeneration over time.

## Endogenous Oligodendrocyte Replacement and Remyelination Following SCI

It is well-known that spontaneous remyelination occurs naturally after CNS injury (Gensert and Goldman, 1997; Salgado-Ceballos et al., 1998). However, the extent and quality of remyelination is limited following injury resulting in limited reorganization of nodes of Ranvier and continued axonal dysfunction (Nashmi et al., 2000; Karimi-Abdolrezaee et al., 2004, 2006). In rat compressive chronic SCI, we found considerable number of chronically injured axons in the rim of white matter that exhibited aberrant distribution of Kv1.1, Kv1.2, and Caspr along the paranodal and juxtaparanodal regions, an established characteristic of axonal demyelination or dysmyelination (Nashmi et al., 2000; Karimi-Abdolrezaee et al., 2004). Additionally, electron micrographs of the injured white matter showed that the spontaneous remyelination after SCI is suboptimal and incomplete as the newly formed myelin around the injured axons is thinner than normally myelinated axons (Nashmi and Fehlings, 2001; Karimi-Abdolrezaee et al., 2006). Considerable evidence over the past years has uncovered that failure of the injured and diseased spinal cord for adequate remyelination is attributed to multiple factors that include (1) the limited replacement of myelinating oligodendrocytes by spinal cord progenitor cells (Mothe and Tator, 2005; Meletis et al., 2008; Barnabe-Heider et al., 2010; Karimi-Abdolrezaee et al., 2012; Gauthier et al., 2013), (2) insufficient levels of key growth factors for oligodendrocyte maturation and myelination (Kakinuma et al., 2004; Almad et al., 2011; Gauthier et al., 2013), (3) inadequate clearance of myelin debris that interferes with the process of axonal ensheathment and remyelination (Naumann et al., 2003; Miron et al., 2011; Lampron et al., 2015), (4) inhibitory factors mainly driven by activated glia that inhibit migration and maturation of OPCs, differentiation of NPCs to oligodendrocytes, and axonal ensheathment (Larsen et al., 2003; Kuhlmann et al., 2008; Karimi-Abdolrezaee et al., 2010; Lukovic et al., 2015). In the following sections, we will discuss endogenous mechanisms of remyelination and the role of injury microenvironment in modulating the replacement of oligodendrocytes and axonal remyelination.

### Replacement of New Myelinating Cells Following SCI

Spinal cord injury results in loss of oligodendrocyte population acutely due to necrosis caused by the primary tissue damage (Almad et al., 2011). However, oligodendrocyte cell loss continues progressively through apoptosis-mediated cell death at subacute and chronic stages of SCI (Casha et al., 2001; Grossman et al., 2001; Almad et al., 2011). Evidence shows that oligodendrocytes are highly susceptible to cell death even after moderate contusive injury resulting in the loss of over 90% of the oligodendrocytes at the lesion epicenter by seven days after injury (McTigue et al., 2001). Interestingly, apoptotic oligodendrocyte death is also observed chronically along the long fiber tracts as a consequence of axonal degeneration and loss of trophic support from axons (Crowe et al., 1997). Similar process has been observed chronically in primate models of contusive SCI (Crowe et al., 1997) and in human SCI (Guest et al., 2005). Multiple secondary injury mechanisms contribute to oligodendrocyte loss in SCI including digestion by proteolytic enzymes released from damaged cells and toxic blood components (Juliet et al., 2009), ischemic damage, and oxidative stress (Thorburne and Juurlink, 1996; McAdoo et al., 1999; Jana and Pahan, 2007), glutamate and ATP mediated excitotoxicity (Wang et al., 2004; Xu et al., 2004; Gudz et al., 2006; Matute et al., 2007), pro-inflammatory cytokines released from infiltrated neutrophils and lymphocytes (Antel et al., 1994; Popovich et al., 1997; Takahashi et al., 2003; Demjen et al., 2004; Pineau and Lacroix, 2007; Donnelly and Popovich, 2008; Kanno et al., 2009) and autophagy (Kanno et al., 2009).

Despite extensive cell death, new oligodendrocytes form and remyelination occurs spontaneously following SCI and demyelinating CNS diseases (Chari, 2007; Zawadzka et al., 2010). Mature oligodendrocytes are post-mitotic and unable to contribute to cell renewal (Keirstead and Blakemore, 1997). However, the spinal cord harbors a population of adult OPCs that contribute to oligodendrocyte replacement following injury (McTigue et al., 2001; Zawadzka et al., 2010). These OPCs can be identified by the expression of platelet derived growth factor receptor α (PDGFR-α) and NG2 proteoglycan (Levine and Nishiyama, 1996; Keirstead et al., 1998; McTigue et al., 2001; Schonberg et al., 2007). Recent findings have shown that resident adult spinal cord OPCs become activated, and change their gene transcription pattern resembling immature OPCs (Moyon et al., 2015). OPCs differentiate into myelinating oligodendrocytes and remyelinate spared axons following demyelination (Gensert and Goldman, 1997; McTigue et al., 2001; Hesp et al., 2015). In addition to OPCs, the spinal cord also contains a population of endogenous NPCs, which is known to contribute to oligodendrocyte replacement following injury (Horky et al., 2006; Meletis et al., 2008; Barnabe-Heider et al., 2010; Karimi-Abdolrezaee et al., 2012). These NPCs exist in the ependymal layer of the intact spinal cord (Weiss et al., 1996; Barnabe-Heider et al., 2010). In adulthood and under normal conditions, NPCs are latent and their activity is mainly to maintain their own population through self-renewal (Meletis et al., 2008; Barnabe-Heider et al., 2010). However, upon injury, they become activated and migrate to the site of injury where they can generate new glial cells (Horner et al., 2000; Barnabe-Heider et al., 2010). Studies by our group and others have demonstrated that activated NPCs predominantly differentiate into astrocytes after SCI, with limited number differentiating into new oligodendrocytes (Mothe and Tator, 2005; Meletis et al., 2008; Barnabe-Heider et al., 2010; Karimi-Abdolrezaee et al., 2012). Moreover, the newly formed NPCs derived astrocytes contribute to glial scar formation following SCI (Meletis et al., 2008; Sabelstrom et al., 2013).

Recent studies have shown that limited ability of NPCs for oligodendrocyte differentiation in SCI milieu may be attributed to unavailability, or modified expression of essential growth factors for oligodendrocyte development (Karimi-Abdolrezaee et al., 2006; Gauthier et al., 2013). Several studies have addressed this possibility by administering growth factors to optimize the post-SCI microenvironment to support survival and differentiation of transplanted and endogenous NPCs into oligodendrocytes as well as remyelination (Karimi-Abdolrezaee et al., 2006; Ohori et al., 2006; Furusho et al., 2012; Gauthier et al., 2013). In our NPC transplantation studies in subacute and chronic SCI, sustained infusion of a cocktail of growth factors including EGF, bFGF, and PDGF-AA was able to significantly enhance the long-term survival of NPCs in the injured spinal cord (Karimi-Abdolrezaee et al., 2006, 2010). Importantly, transplanted NPCs were able to survive and integrate within the host tissue and differentiate into mature myelinating oligodendrocytes and remyelinated axons (Karimi-Abdolrezaee et al., 2006). EGF and bFGF are known to play essential role in NPC survival and proliferation (Weiss et al., 1996). *In vivo* delivery of these growth factors into animal models of contusive SCI was associated with increased proliferation in ependymal layer where NPCs reside. However, despite increased proliferative activity, no significant change in oligodendrogenesis were seen, which could be due to the lack of PDGF-AA in this growth factor regimen (Kojima and Tator, 2000, 2002). PDGF-AA promotes the proliferation of glial progenitor cells and can trigger differentiation and survival of newly formed oligodendrocytes (Raff et al., 1988; Butt et al., 1997). Moreover, bFGF in synergy with PDGF can regulate proliferation of adult OPCs (Lachapelle et al., 2002; Frost et al., 2003). Interestingly, a developmental study on FGF receptor 1 and 2 double knockout mice (Fgfr1^-/-^, Fgfr2^-/-^) showed normal OPC proliferation, differentiation and initiation of myelination. However, these FGF receptor null animals demonstrated defective myelin thickening during postnatal period and remained defective throughout their adulthood (Furusho et al., 2012). This evidence suggests that FGF signaling can regulate myelin sheath thickness (Furusho et al., 2012).

Mash1, a transcription factor known to promote neural differentiation, have also been implicated in endogenous oligodendrocyte differentiation (Parras et al., 2004; Ohori et al., 2006). Retroviral induction of Mash1 expression in endogenous spinal cord NPCs following SCI resulted in increased oligodendrocyte differentiation and formation of new oligodendrocyte progenitor cells following a complete transection rat model of SCI. Although a small number of new Mash1 expressing oligodendrocytes expressed markers of mature oligodendrocytes, the majority of these cells remained NG2+ expressing progenitor cells and did not fully mature into myelinating oligodendrocytes even weeks after injury (Ohori et al., 2006).

Neuregulin-1 (Nrg-1) is another growth factor known to promote OPCs survival, migration, and differentiation into mature myelinating oligodendrocytes (Vartanian et al., 1999; Miller, 2002). Nrg-1 is known to play essential roles in oligodendrocyte and SC myelination (Brinkmann et al., 2008). Our group has recently demonstrated that the rapid and long lasting downregulation of Nrg-1 following contusive SCI is an underlying cause of inadequate oligodendrocyte differentiation (Gauthier et al., 2013). Restoring the reduced levels of Nrg-1 in the injured spinal cord enhanced tissue preservation, oligodendrocyte differentiation of spinal cord NPCs, and increased oligodendrocyte and axonal survival following SCI (Gauthier et al., 2013). Collectively, these studies show the necessity of micro-environmental optimizations in order to improve endogenous and exogenous replacement of oligodendrocytes and axon remyelination following SCI.

### Inhibition of Remyelination after SCI

Current evidence shows that remyelination is additionally limited by inhibitory modifications in the post-SCI niche caused by secondary injury mechanisms particularly in chronic SCI (Larsen et al., 2003; Kuhlmann et al., 2008; Karimi-Abdolrezaee et al., 2010; Lau et al., 2012; Lukovic et al., 2015). Newly formed oligodendrocytes often fail to fully ensheath and myelinate the injured spared axons following injury resulting in incomplete remyelination (Salgado-Ceballos et al., 1998). These inhibitory signals are primarily associated with myelin debris, activated glial cells, and infiltrating leukocytes following injury (Ji et al., 2006; Kotter et al., 2006; Fancy et al., 2009; Pohl et al., 2011; Plemel et al., 2013; Smith et al., 2014; Tepavcevic et al., 2014).

Presence of myelin debris and insufficient clearance by microglia and macrophages contributes to incomplete remyelination by inhibiting OPCs differentiation and maturation *in vitro* and *in vivo* (Kotter et al., 2006; Nave, 2010; Plemel et al., 2014). Recent *in vitro* studies by Plemel et al. (2013) indicate that exposure to myelin debris prevents OPCs maturation and their transition to a myelinating phenotype (Plemel et al., 2013). This was demonstrated by a significant decrease in the number of mature oligodendrocytes and was accompanied by increased expression of two proteins, namely inhibitor of differentiation (ID) 2 and ID4 that are known to block oligodendrocyte maturation (Plemel et al., 2013). It has been shown that myelin clearance and remyelination become less sufficient with aging due to changes in macrophage secretory and phagocytic activity (Shields et al., 1999; Miron and Franklin, 2014). A study using a technique known as “heterochronic parabiosis” where the circulation of a young animal is infused into an older animal, demonstrated improvements in remyelination in the old animal which is presumably due to better functioning of young circulating monocytes for myelin debris clearance (Miron and Franklin, 2014). Myelin debris is a potent inhibitory component of injured spinal cord that impairs regeneration and remyelination. Thus, proper myelin clearance is an important step for remyelination process (Kotter et al., 2006).

Other molecules and pathways known to inhibit myelination include LINGO (leucine rich repeat and Ig domain-containing, Nogo receptor interacting protein), Wnt signaling, and Semaphorin 3A (Sema3A) (Ji et al., 2006; Fancy et al., 2009; Ye et al., 2009; Syed et al., 2011; Boyd et al., 2013). LINGO-1 is a component of Nogo receptor signaling complex (Ji et al., 2006). In a hemisection model of SCI, application of LINGO-1 antagonist (LINGO-1-Fc) promoted functional recovery (Ji et al., 2006). Dysregulation of Wnt signaling in OPCs also inhibits myelination during development and repair (Fancy et al., 2009; Ye et al., 2009). Wnt signaling is activated in differentiating OPCs following chemically induced demyelination and in samples from MS patients (Fancy et al., 2009). Following demyelination, upregulation of T-cell factor 4 (tcf4), a Wnt pathway mediator, is significantly upregulated in differentiating OPCs and inhibit oligodendrocyte maturation and myelination (Fancy et al., 2011). Similarly, Sema3A negatively affects OPC maturation and recruitment in demyelinating conditions (Syed et al., 2011). Its level increases significantly after SCI, reaching its peak at one week following injury (Kaneko et al., 2006). Increased expression of Sema3A has also been observed in MS and experimental demyelination models (Piaton et al., 2011; Boyd et al., 2013). Sema3A overexpression delays recruitment of OPCs to the demyelination site through a chemo-repulsive mechanism (Piaton et al., 2011). Use of Sema3A inhibitor improved tissue preservation, remyelination and functional recovery following SCI (Kaneko et al., 2006).

Collectively, these findings demonstrate that endogenous remyelination was impeded by the inhibitory microenvironment following injury and activated astrocytes and microglia/macrophages seem to play pivotal roles in this inhibition. We will discuss recent studies on the role of resident glial cells and peripherally recruited immune cells in modulating oligodendrocyte replacement and remyelination following CNS injury.

## Role of Glial Cells in Myelination

### Astrocytes and CNS Myelination

Astrocytes play critical role in several aspects of myelination in pathologic CNS including clearance of myelin debris, modulating the activity of oligodendrocytes, myelin maintenance, and renewal (Sorensen et al., 2002; Kakinuma et al., 2004; Iacobas and Iacobas, 2010; Moore et al., 2011; Siebert and Osterhout, 2011; Schulz et al., 2012; Bracchi-Ricard et al., 2013; Pendleton et al., 2013; Skripuletz et al., 2013; Brambilla et al., 2014). Using a cuprizone model of rodent demyelination, Skripuletz et al. (2013) demonstrated that astrocytes contribute to the clearance of myelin debris by inducing the recruitment of microglia into demyelinated lesion sites (Skripuletz et al., 2013). Astrocytes impose their modulatory effects through upregulation of CXCL10, a chemokine that is known to play a role in T-cell chemoattraction in CNS autoimmune disorders such as MS (Sorensen et al., 2002).

Intercellular connections between astrocytes and oligodendrocytes are critical for the proper physiology of oligodendrocytes. While there are no gap junctions between oligodendrocytes themselves, they are connected to astrocytes through gap junctions, which make oligodendrocytes indirectly interconnected (Nagy et al., 1997, 2001; Menichella et al., 2003). Evidence shows that gap junctions are essential for proper myelin physiology in the CNS (Menichella et al., 2003). Four different types of connexins have been identified in oligodendrocytes (Cx29, 32, 45, 47). Cx29, Cx32, and Cx47 are known to be expressed by oligodendrocytes that in conjunction with Cx26, 30, 43 on astrocytes, form the astrocyte-oligodendrocyte gap junction complex (Scherer et al., 1995; Altevogt et al., 2002; Menichella et al., 2003; Nagy et al., 2007). Double knockout mice models lacking Cx47 and Cx32 die postnatally due to severe apoptotic oligodendrocyte death, hypomyelination, and axonal degeneration (Menichella et al., 2003). This evidence suggests a critical role for astrocyte and oligodendrocytes inter-cellular signaling in myelin physiology.

Astrocytes provide trophic support to oligodendrocytes by producing growth factors. In an ethidium bromide (EB) induced rat model of spinal cord demyelination, Talbott et al. (2005) were able to show despite recruitment of OPCs to the site of injury, they failed to mature and remyelinate axons in the areas that astrocytes were absent. Astrocytes are known to produce PDGF and LIF, which are supportive for oligodendrocyte survival at progenitor and mature stages, respectively (Barres et al., 1993; Gard et al., 1995).

While supportive of myelination in the normal CNS, astrocytes can play detrimental roles in CNS remyelination following pathology (Rosen et al., 1989; Kakinuma et al., 2004; Pendleton et al., 2013; Brambilla et al., 2014; Hammond et al., 2014). Astrocytes contribute substantially to the extracellular matrix of the CNS. Following injury, they are activated and form a glial scar, which is inhibitory to the repair and regeneration of the CNS. The inhibitory influence of scar is mediated mainly through chondroitin sulfate proteoglycans (CSPGs), which have known inhibitory effects on axonal regeneration, axonal conduction, remyelination, and cellular therapies in SCI (Massey et al., 2008; Karimi-Abdolrezaee et al., 2010, 2012; Haylock-Jacobs et al., 2011; Lau et al., 2012; Cua et al., 2013; Cregg et al., 2014; for review see Dyck and Karimi-Abdolrezaee, 2015). Our recent evidence shows that CSPGs inhibit the ability of NPCs to proliferate, spread their cell processes, survive and differentiate into oligodendrocytes (Dyck et al., 2015). Degradation of CSPGs with chondroitinase ABC promotes oligodendrocyte differentiation and myelination of both transplanted NPCs (Karimi-Abdolrezaee et al., 2010) and endogenous precursor cell populations (Karimi-Abdolrezaee et al., 2012). The detrimental effect of CSPGs upregulation by astrocytes is also observed in MS lesion where the CSPGs aggrecan, neurocan, and versican as well as hyaluronan accumulate at the borders of active demyelinating lesions (Back et al., 2005; Chang et al., 2012). *In vivo* and *in vitro* observations have shown that CSPGs limit the ability of OPCs to migrate, mature and myelinate axons (Kuhlmann et al., 2008; Lau et al., 2012; Pendleton et al., 2013). Removal of CSPGs is correlated with enhanced remyelination in MS lesions (Lau et al., 2012). Collectively, these data identify the inhibitory role of activated astrocytes and scar-associated CSPGs, in modulating NPCs and OPCs integration, migration, maturation and myelination in SCI and MS conditions (Dyck and Karimi-Abdolrezaee, 2015). In addition to the inhibitory ECM produced by astrocytes, reactive astrocytes can also be detrimental to remyelination in demyelinated CNS through the secretion of Endothelin-1 (Hammond et al., 2014). Endothelin-1 is shown to inhibit the differentiation of OPCs into mature myelinating oligodendrocytes through the activation of Notch signaling.

Taken together, these data demonstrate the complex role of astrocytes in the CNS. The presence of astrocytes is required to produce healthy myelin, however, the detrimental effects of activated astrocytes and their production of inhibitory ECM molecules following injury limits the ability of the CNS in self-repair and axon remyelination. Thus, developing interventions to moderate the inhibitory effects of scar-associated molecules is a vital therapeutic strategy for CNS repair and remyelination following injury.

### Macrophages/Microglia and CNS Myelination

Emerging evidence indicates that macrophages and microglia also play critical roles in modulating demyelination and remyelination through their antigen presenting ability and production of cytokines, chemokines and growth factors (for review see Gordon, 2003; Mosser, 2003; Martinez et al., 2008). After CNS injury or infection, microglia/macrophages undergo phonotypical changes and become polarized into pro-inflammatory “classically activated” M1 or anti-inflammatory “alternatively activated” M2 phenotypes (as reviewed by Mosser, 2003; Martinez et al., 2008; Kigerl et al., 2009). Although both M1 and M2 cell types are activated microglia/macrophages, they play distinct roles in CNS injury and repair. Pro-inflammatory M1 microglia/microphages are characterized by the production of cytokines such as interleukin (IL)- 1β, IL-6, IL-12, tumor necrosis factor (TNF)-α (Mosser, 2003; Cherry et al., 2014; Kroner et al., 2014; Peferoen et al., 2015) and reactive oxygen and nitrogen species such as nitric oxide (NO) (Mosser, 2003; Peferoen et al., 2015). Conversely, M2 microglia/microphages are a source of anti-inflammatory factors such as arginase-1 (Arg-1) and IL-10, which are known for their role in the development of type II adaptive immune responses (Anderson and Mosser, 2002; Miron and Franklin, 2014). Generally, accumulating evidence has identified a pro-regeneration role for M2 microglia/macrophages including a supporting role in overcoming axonal growth inhibition imposed by CSPGs and myelin debris (Kigerl et al., 2009).

A recent study also demonstrated phonotypical changes in macrophages/microglia following lysolecithin-induced demyelination in mice (Miron et al., 2013). Using specific M1/M2 markers, Miron et al. (2013) identified a “switch” from M1 to M2 phenotype following demyelination. This transformation occurred between day 3 to 10 post-lysolecithin-induced demyelination when M1 dominant population of CD68^+^ macrophages/microglia adopted a M2 dominant phenotype identified by Arg-1 expression. This time window was closely correlated with a regenerative stage at which OPCs were recruited to the site of lesion, and differentiated into mature myelinating oligodendrocytes (Miron et al., 2013). Further *in vitro* and *in vivo* investigations confirmed a supportive role for M2 microglia/macrophages in remyelination. Adding M2 conditioned media into OPCs cultures increased oligodendrocyte differentiation and maturation (Miron et al., 2013; Miron and Franklin, 2014). Selective depletion of M1 macrophages by intralesional injection of gadolinium chloride reduced the proliferation rate of OPCs without affecting their migration and remyelination capacity. Interestingly, in a rat model of lysolecithin demyelination, M2 depletion was associated with delayed oligodendrocyte differentiation and nodal reconstruction (Miron et al., 2013) suggesting the pro-myelinating role of M2 macrophages.

Activation of microglia through intraspinal injection of lipopolysaccharide (LPS), a Toll-like receptor-4 (TLR-4) agonist, caused a significant increase in NG2^+^ cell proliferation and oligodendrocyte differentiation. However, activating microglia using intraspinal injection of zymosan, a TLR-2 agonist, showed oligodendrocyte loss without increase in NG2^+^ cell proliferation (Schonberg et al., 2007). Interestingly, the extent of SC remyelination remained unaffected (Kotter et al., 2001). Of note, these studies also revealed that the timing of macrophages response is a key factor as the early presence of the macrophages was important for remyelination while delayed macrophage depletion did not impair remyelination (Kotter et al., 2001).

Several mechanisms have been proposed for the positive role of macrophages in remyelination. Among the main proposed mechanisms are removal of myelin debris (Ousman and David, 2000) and production of growth factors known to promote oligodendrocyte differentiation such as Insulin like Growth Factor (IGF)-1 and TGF-β1 (Hinks and Franklin, 1999; Woodruff and Franklin, 1999; Hsieh et al., 2004). However, recent studies by Kotter et al. in demyelinating models suggest that failure in remyelination is mainly attributed to the loss of macrophages-derived promoting factors rather than their role in myelin clearance since this function can be covered by the resident microglia (Kotter et al., 2001; Miron et al., 2013).

Altogether, evidence indicates that the type of immune response is a determining factor that can promote or inhibit remyelination in demyelinating CNS lesions (as reviewed by Mosser, 2003; Wee Yong, 2010). Accordingly, targeted immunomodulatory strategies rather than complete anti-inflammatory treatments appears to be a more effective strategy for promoting remyelination in autoimmune demyelinating conditions such as MS. Further research was needed to elucidate the mechanisms involved in immune response after demyelinating CNS conditions and the factors that promote remyelination (Jackson et al., 2011).

### Schwann Cells and Spinal Cord Remyelination

Following SCI or demyelination, endogenous SCs invade the injury site and contribute to remyelinating the demyelinated axons (Blakemore, 1975; Bunge et al., 1994; Beattie et al., 1997; Guest et al., 2005). SCs entry to the injured spinal cord occurs during the first week of injury in parallel to clearance of myelin and glial debris by microglia/macrophages (Bunge et al., 1994; Beattie et al., 1997). SCs enter through dorsal funiculi via dorsal root entry zone or lateral funiculi from the rootlets that become adhered to the lateral spinal cord after injury (Jasmin et al., 2000). In chemical models of demyelination in rodents, remyelination by endogenous SCs and oligodendrocytes progress simultaneously and fully myelinate demyelinated axons by 4 weeks following the insult. However, the extent of oligodendrocytes remyelination is smaller when compare to peripheral myelin formed by SCs and is restricted to the edges of the lesion (Blakemore, 1975). The limited degree of oligodendrocyte remyelination has been attributed to the absence of astrocytes in chemically demyelinated lesions, as oligodendrocytes are dependent on astrocytes for remyelination (Blakemore, 1975; Blakemore and Patterson, 1975).

Entry of SCs to the injured spinal cord is normally limited by glia limitans formed by astrocytes. Following insult, SCs invade the spinal cord through the regions where glia limitans is disrupted. When the glial limitans is re-established by astrocytes, SC invasion becomes progressively limited (Blakemore, 1976). Interestingly, studies have shown that oligodendrocytes gradually replace SCs in remyelinated axons and the transition from peripheral to central myelination occurs without any loss of function (Jasmin et al., 2000). However, other studies showed that SCs persist even chronically following SCI and continue myelinating axons (Hill et al., 2003). Subsequent studies investigated the transition from SCs to oligodendrocytes remyelination and found no change in SC myelination despite increasing oligodendrocyte myelination in EB and radiation (X-EB) demyelination model (Gilson and Blakemore, 2002). There is also evidence that transplantation of OECs, SCs, and bone marrow stromal cells can promote migration of SCs from dorsal roots into the injury site (Hill et al., 2006; Biernaskie et al., 2007). Therefore; enhanced remyelination or other beneficial effects observed after cell transplantation can be partially attributed to migrating SCs particularly in studies with poor survival of transplanted cells. This evidence suggests that SCs serve as emergency responders and protect demyelinated spinal cord axons at the time when oligodendrocytes are unable to remyelinate efficiently.

## Cell-Based Strategies for Remyelination after SCI

Over the past decade, efforts from our group and others have been made to enhance oligodendrocyte replacement after SCI by cell transplantation or activating endogenous stem/progenitor cells (Hofstetter et al., 2005; Keirstead et al., 2005; Karimi-Abdolrezaee et al., 2006, 2010, 2012; Parr et al., 2008; Gauthier et al., 2013; Sparling et al., 2015). Cell transplantation in particular has shown promising results in enhancing SCI repair through multiple mechanisms including cell replacement, trophic support, immunomodulation, and remyelination (Ogawa et al., 2002; Okano et al., 2003; Cummings et al., 2005; Hofstetter et al., 2005; Karimi-Abdolrezaee et al., 2006, 2010; Rossi et al., 2010; Tetzlaff et al., 2011; Vaquero and Zurita, 2011; Hawryluk et al., 2012, 2014). Using different cell types (**Table 1**), these studies have suggested that remyelination is a key mechanism in promoting functional recovery following SCI and demyelinating conditions (Karimi-Abdolrezaee et al., 2006; Sasaki et al., 2006; Eftekharpour et al., 2007; Yasuda et al., 2011; Hawryluk et al., 2014).

**Table 1:** List of selected cell therapies for promoting remyelination following spinal cord injury (SCI) and multiple sclerosis (MS).

Reference	Cell type	Injury model	Outcome
Hofstetter et al. (2005)	Adult rat spinal cord NPCs (Naïve or transduced to express Neurogenin-2)	Thoracic contusive rat SCI, (Subacute)	Increased myelination and white matter sparing in Ngn2-NPC group. Improved BBB and grid-walking in Ngn2-NPC group.
Keirstead et al. (2005)	hESC derived OPCs	Thoracic contusive rat SCI, (Subacute and chronic)	Significant remyelination occurred in subacute OPCs transplantation. Improved functional recovery was observed after subacute transplantation.
Karimi-Abdolrezaee et al. (2006)	Adult brain NPCs + growth factor cocktail	Thoracic compressive SCI (subacute and chronic)	Significant oligodendrocyte replacement and remyelination in subacute transplantation (2 weeks post-injury). Significant functional improvement in subacute therapy (BBB, grid-walking and footprint analysis). Chronic transplantation was not successful (8 weeks post-injury).
Karimi-Abdolrezaee et al. (2010)	Adult brain NPCs + ChABC and growth factors	Thoracic compressive SCI (chronic)	Significant improvement in remyelination and functional recovery in transplanted animals.
Eftekharpour et al. (2007)	Adult brain NPCs	adult *Shiverer* mice	Myelination of chronically dysmyelinated axons happened in transplanted group. Reconstruction of nodes of Ranvier and enhanced axonal conduction.
Sasaki et al. (2006)	Adult OECs from	Adult rat model of spinal cord X-EB demyelination	Transplanted OECs integrated with host tissue and remyelinated axons. Nodes of Ranvier were reconstructed and conduction velocity was significantly restored.
Yasuda et al. (2011)	Adult NPCs derived from wild-type or *Shiverer* mice	Thoracic contusive SCI in adult NOD/SCID mice	Both cell types survived after transplantation and exhibited similar differentiation potential. Only wild-type NPC group demonstrated preserved or enhanced myelination and significant functional and electrophysiological recovery.
Hawryluk et al. (2014)	Adult mice brain NPCs from wild-type or *Shiverer* mice	Rat thoracic contusive SCI using 23 g clip compression	*Shiverer* NPC transplanted group showed no significant remyelination and no significant change in functional recovery while wild-type NPC transplanted group demonstrated significant remyelination by transplanted cells and significant functional recovery.
Kumagai et al. (2009)	Primary and secondary neurospheres (PNS and SNS) from CCV-ES cell line derived NPCS	Mouse thoracic contusive SCI	PNS and SNS survived in host tissue. Transplanted SNS but not PNS showed remyelination, axonal regeneration and functional recovery.
Sharp et al. (2010)	hESC derived OPCs	Rat cervical midline contusive SCI	Significant white and gray matter sparing. Significantly higher properly oligodendrocyte remyelinated axons.
Sun et al. (2013)	mESC derived OPCs	Radiation induced rat cervical spinal cord demyelination	Transplanted cells survived and integrated into the host tissue, migrated to the injured tissue and differentiated into oligodendrocytes. Improvement of forelimb locomotor function.
Windrem et al. (2008)	Human GRPs	Neonatal *Shiverer* mice cross bred with immune-deficient *rag2* null mice	Multifocal anterior and posterior fossa delivery of hGRPs showed significant improvement in survival, neurological function and seizure frequency in neonatal *Shiverer* mice.
Hwang et al. (2009)	hNPCs expressing Olig2	Adult rat contusive SCI	Transplanted animals exhibited enhanced myelination in white matter and improved functional recovery.
Cao et al. (2010)	CNTF expressing adult rat spinal cord OPCs	Thoracic contusive SCI	Enhanced remyelination and functional recovery in transplanted animals.
Utzschneider et al. (1994)	Adult spinal cord glial cells	Neonatal congenitally myelin deficient rats	Improvement in conduction velocity of axons in transplanted region was observed.
Walczak et al. (2011)	Human glial restricted progenitors	Adult rat chemical focal demyelination model and neonatal rag2^-/-^*shiverer* mice	Transplanted rats showed preserved electrophysiological conduction across spinal cord. Despite extensive remyelination in neonatal *Shiverer* mice marginal myelination observed in transplanted adult rats.
Totoiu et al. (2004)	Neonatal mouse glial committed progenitors	Mouse HMV induced CNS demyelination	Remyelination in transplanted animals was evident with significant axonal preservation and locomotor recovery.
Hatch et al. (2009)	hESC derived OPCs	JHMV induced CNS demyelination	Transplanted cells failed to survive beyond 2 weeks. Focal remyelination and subtle functional recovery was observed that was attributed to inflammatory modulation and trophic support provided by transplanted cells.
Piao et al. (2015)	hESC derived OPCs	Athymic nude rat model of radiation induced brain demyelination	Transplantation to several spots along cerebellum and forebrain showed significant remyelination and cognitive and motor improvement.
Imaizumi et al. (2000)	Adult OECs or Schwann cells (SCs) from pigs expressing human complement inhibitory protein, CD59	Adult rat dorsal column transection	Remyelination of demyelinated axons was observed with improvement in conduction velocity in transplanted animals.
Radtke et al. (2004)	Adult porcine OECs	EBr or lysophosphatidyl choline induced demyelination in African green monkey spinal cord	Transplanted cells integrated with host tissue and remyelinated axons.
Boyd et al. (2004)	LacZ expressing OECs injected into the cystic cavity	Rat model of thoracic contusive SCI	Transplanted cells did not migrate from the injection site. SCs migrated into the cystic cavity. No direct evidence of remyelination by LacZ labeled OECs was observed. OECs mainly enveloped groups of axons myelinated by SCs.
Sasaki et al. (2011)	Rat OECs	Rat model of X-EB induced spinal cord demyelination (acute and subacute)	Transplanted cells integrated with host tissue and remyelinated axons. Reconstruction of nodes of Ranvier and improved axonal conduction velocity were achieved. Remyelination was higher in acutely transplanted group.
Tsuji et al. (2010)	MEF-iPSCs derived NPCs	Mouse model of contusive SCI	Transplanted cells showed multilineage differentiation into oligodendrocytes, astrocytes and neurons.
Wang et al. (2013)	Human skin derived iPSC derived OPCs	*Shiverer* mice neonatal brain	Transplanted cells integrated with host tissue and differentiated into astrocytes and MBP expressing oligodendrocytes. *Shiverer* axons were remyelinated and node of Ranvier was reconstructed. No tumorigenicity was observed. Increased lifespan of *Shiverer* mice was observed.
Nori et al. (2011)	hiPSC derived neurospheres	Thoracic contusive SCI in NOD-SCID mice	Grafted cells differentiated into neurons, astrocytes and oligodendrocytes. Myelin content of the tissue was increased with significantly better functional recovery in transplanted group.
Kobayashi et al. (2012)	hiPSC-NS/PCs	Adult common marmoset primate model of contusive SCI	Improved functional recovery in open field, bar grip and cage climbing tests in transplanted group.

### Neural Stem and Progenitor Cells

**FIGURE 2:**
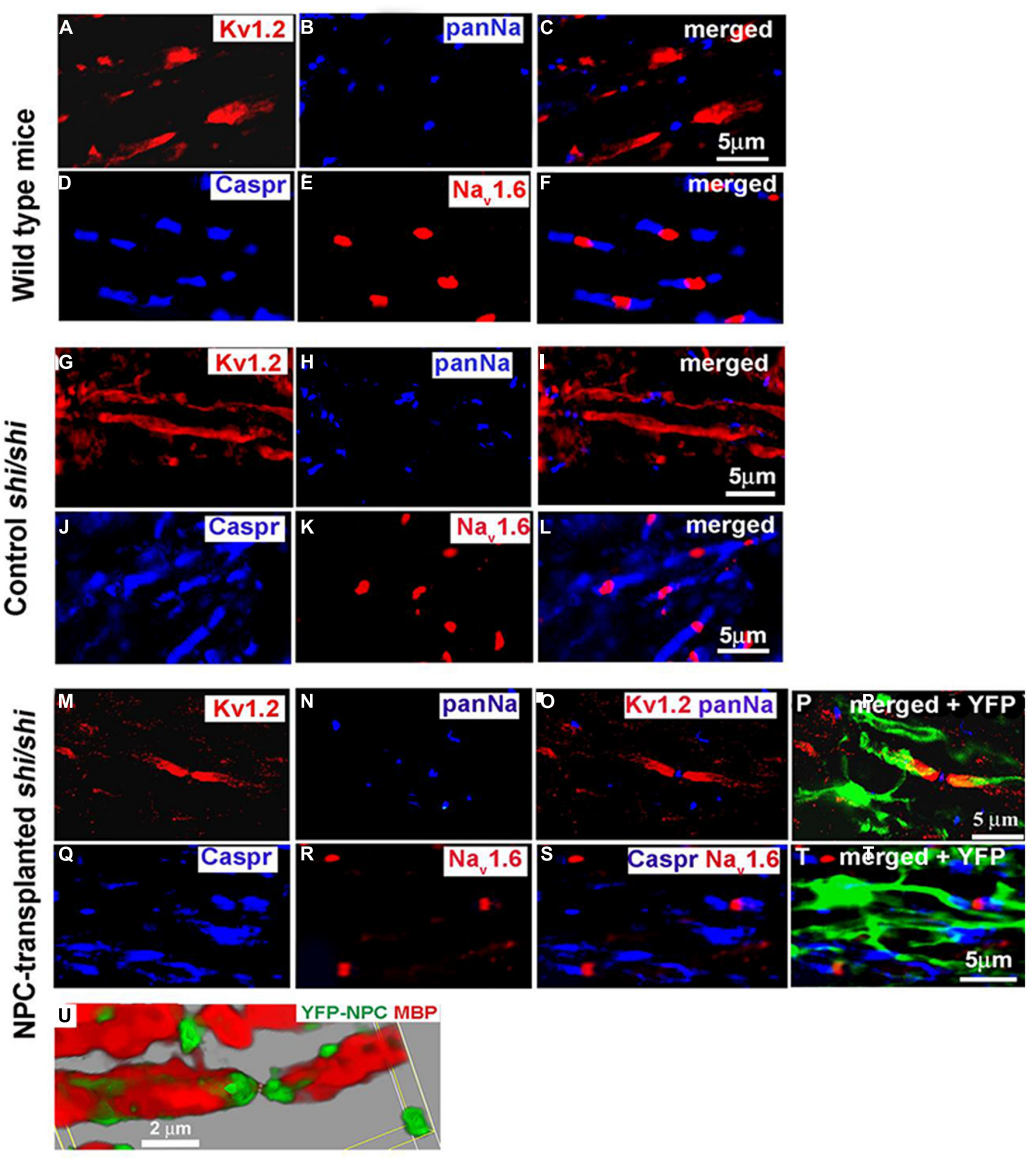
**Transplanted adult NPCs (aNPCs) promote the aggregation of K^+^ channels and the formation of nodes of Ranvier in the spinal cord axons of *shi/shi* mice.** Confocal immunostaining of Kv1.2 subunits (red) and pan-Na^+^ hannels (blue) in the spinal cord of wild-type mice **(A–C)**, control *shi/shi* mice **(G–I)**, and transplanted *shi/shi* mice **(M–P)** is depicted. Kv1.2 subunits were clearly localized to the juxtaparanodal regions of wild-type spinal cord axons **(A–C)**, confirmed with nodal pan-Na^+^ immunostaining. In *shi/shi* mice, Kv1.2 immunostaining was abnormally distributed along the axonal internodes **(G–I)**, but Na^+^ clusters were observed as aberrant nodal aggregates. Six weeks after aNPCs transplantation, spinal cord segments of *shi/shi* mice showed restoration of Kv1.2 subunit clusters **(M–P)**. YFP-positive processes of transplanted aNPCs were observed in close association with axons containing restored K^+^ channels aggregates **(P)**. Nodal localization of Na^+^ channels was further confirmed using Nav1.6 (red) immunostaining in wild-type **(D–F)**, control *shi/shi* **(J–L)**, and transplanted *shi/shi* **(Q–T)**. Caspr (blue) immunostaining was used to identify the paranodal area. A 3D reconstruction clearly shows a node of Ranvier that is bordered by an MBP-expressing NPC derived oligodendrocyte. Note that the processes of YFP-labeled oligodendrocytes avoid the nodal region **(U)**. From Eftekharpour et al. (2007).

Potential of transplanting NPCs or glial progenitor cells in promoting remyelination has been explored in a wide variety of pathological conditions such as SCI, genetically myelin deficient rodent models, and MS (Karimi-Abdolrezaee et al., 2006; Eftekharpour et al., 2007; Cao et al., 2010; Karimi-Abdolrezaee et al., 2010; Sharp et al., 2010; All et al., 2012; Sun et al., 2013; Hawryluk et al., 2014). These studies have collectively demonstrated the ability of transplanted NPCs to differentiate into myelinating oligodendrocytes and ensheath demyelinated axons. Our studies in mutant *Shiverer* mice and rat SCI revealed that NPC-derived oligodendrocytes integrate with demyelinated and dysmyelinated axons and successfully remyelinate them (Karimi-Abdolrezaee et al., 2006; Eftekharpour et al., 2007) (**Figure 2**). When we transplanted brain-derived NPCs into the spinal cord of subacutely injured rats, we found that survival and oligodendrocyte differentiation of NPCs was limited in the injury microenvironment (Karimi-Abdolrezaee et al., 2006; Eftekharpour et al., 2007) Improving the microenvironment of engrafted NPCs with a cocktail of growth factors (EGF, bFGF, PDGF-AA) considerably promoted their long-term survival, tissue integration, and oligodendrocyte differentiation and remyelination (Karimi-Abdolrezaee et al., 2006, 2010). Importantly, in adult *Shiverer* mice transplanted with NPC, we found evidence of myelination and normal reconstruction of the node of Ranvier in chronically dysmyelinated axons (Eftekharpour et al., 2007). In the areas that NPC-derived oligodendrocytes enwrapped and myelinated the *Shiverer* axons, restoration of a normal configuration of paranodal and juxtaparanodal structures was achieved accompanied by improved axonal function in myelinated axons (**Figure 2**). Similarly, in our rat SCI studies, evidence of NPC-derived remyelination was confirmed with immunoelectron microscopy against YFP expression in transplanted YFP-NPCs (Karimi-Abdolrezaee et al., 2006). Of note, in these studies, transplantation of NPCs resulted in improved locomotor recovery evident by significant improvements in BBB and grid walking test as well as foot print analysis (Karimi-Abdolrezaee et al., 2006).

Subsequent studies by Windrem et al. (2008) demonstrated similar outcomes following transplantation of human glial progenitor cells into the brain of immune-deficient neonatal *Shiverer* mice. In this study, transplanted cells successfully differentiated into myelinating oligodendrocyte and functionally myelinated the dysmyelinated host axons in forebrain and brainstem (Windrem et al., 2008). In agreement with our studies, immunohistological and electrophysiological evidence revealed reconstruction of the node of Ranvier in transplanted neonatal *Shiverer* mice and restoration of transcallosal conduction velocity (Windrem et al., 2008). Moreover, transplanted mice showed increased lifespan and decreased seizure rate, which is frequently seen in *Shiverer* mice (Windrem et al., 2008). Collectively, these studies provided proof-of-concept evidence that NPC-derived oligodendrocytes can functionally remyelinate chronically demyelinated axons in SCI and demyelinating lesions.

Recent studies have provided further evidence that implicates remyelination as a key mechanism for neurological improvement observed after transplantation of NPCs in models of SCI (Yasuda et al., 2011; Hawryluk et al., 2014). Yasuda et al. (2011) transplanted *Shiverer-*derived NPCs that lack the capacity for myelination into the injured spinal cord of NOD/SCID immune-deficient mice. Neuroanatomical, functional, and electrophysiological analyses demonstrated better outcomes in the injured mice transplanted with wild-type NPCs compared to the mice that received *Shiverer* NPCs (Yasuda et al., 2011). This work and similar study by Hawryluk et al. (2014) suggest that remyelination is a key mechanism by which NPCs contributes to the functional recovery following transplantation in SCI.

### Genetically Modified NPCs for Remyelination

As mentioned earlier, in the post-SCI microenvironment, transplanted stem cells exhibit limited capacity for survival and migration and they primarily differentiate into astrocytes at the expense of oligodendrocytes and neurons (Hofstetter et al., 2005; Karimi-Abdolrezaee et al., 2006, 2010). Genetic modifications in NPCs have been made to induce expression of specific classes of transcription or growth factors in order to enhance their survival and differentiation or modulate the hostile microenvironment of SCI (Coumans, 2001; Linker et al., 2002; Murray et al., 2002; Hwang et al., 2009; Yang et al., 2009; Cao et al., 2010; Zhang et al., 2012). Using this approach, impact of oligodendrocyte transcription factor, Olig2 has been investigated in myelin physiology and repair. Olig2 is known to play important role in oligodendrocyte differentiation during development (Takebayashia et al., 2000). Using a contusive model of rat SCI, Hwang et al. (2009) demonstrated that retroviral overexpression of Olig2 in transplanted F3 human NPCs enhanced oligodendrocyte replacement accompanied by a significant increase in white matter sparing, decreased lesion size, increased myelin thickness and improved locomotor recovery compared to non-transduced F3 NPCs. In another study, using transgenic mice overexpressing Olig2 in SOX10^+^ oligodendrocytes, Wegener et al. (2015) confirmed that Olig2 overexpression enhanced OPC differentiation, maturation, and migration as well as remyelination following chemical demyelination. Studying demyelinated lesions in human MS brain samples revealed that Olig2^+^ remyelinating oligodendrocytes are more abundantly present in active lesion borders compared to chronic plaques (Wegener et al., 2015). This evidence identifies a role for Olig2 in promoting oligodendrocyte differentiation and remyelination.

A neuroprotective role for ciliary neurotrophic factor (CNTF) in enhancing oligodendrocyte differentiation and maturation has been established in the CNS and PNS (Hagg and Varon, 1993; Linker et al., 2002; Naumann et al., 2003). Genetic modifications in adult rat spinal cord OPCs to express CNTF attenuated oligodendrocyte apoptosis resulting in improved survival, maturation and myelinating capacity of OPCs (Cao et al., 2010). CNTF expressing OPCs transplanted rats also showed significant improvements in their axonal signal conduction and hind limb locomotor recovery compared to the group that received normal OPCs (Cao et al., 2010).

Altogether, current evidence suggests that NPCs can be engineered to act as environmental modulators in addition to their role in cell replacement. This strategy presents a therapeutic avenue for improving microenvironment and optimizing the outcome of cell transplantation in CNS trauma and demyelinating diseases.

### Glial-Restricted and OPCs

Due to challenges with oligodendrocyte differentiation of NPCs, transplantation of differentiated glial-restricted precursors (GRP) and OPCs has been pursued in SCI and demyelinating conditions (Utzschneider et al., 1994; Keirstead et al., 2005; Walczak et al., 2011; Sun et al., 2013). Early studies demonstrated the potential of bi-potential O2A progenitor cells for differentiation into oligodendrocytes or type 2 astrocytes (De Los Monteros et al., 1993). When postnatal O2A progenitor cells, genetically modified to express β-galactosidase, were transplanted into the spinal cord following x-ray induced demyelination, they showed the capacity to form oligodendrocytes and successfully remyelinated axons in demyelinated lesion (Groves et al., 1993). Interestingly, no evidence of astrocyte differentiation was observed after transplantation of O2A progenitors (Groves et al., 1993) suggesting the influence of demyelinated host tissue on cell fate specification of transplanted cells. Successful remyelination also achieved following transplantation of brain-derived mouse GRPs into the spinal cord of mice with virally induced demyelination lesion that was associated with improved functional recovery (Totoiu et al., 2004).

Application of OPCs has also shown promising results in supporting remyelination. The potential of human ESC derived OPCs for remyelination has been evaluated in animal models of SCI and demyelination (Keirstead et al., 2005; Sharp et al., 2010; Piao et al., 2015). These studies uncovered that subacute stage of injury is the optimal time window for OPCs transplantation in the context of traumatic SCI. Keirstead et al. (2005) compared remyelination and functional recovery following transplantation of hESC derived OPCs at 7 days (subacute) and 10 months (chronic) after contusive SCI in rats. Subacute transplantation was associated with successful remyelination by transplanted OPCs and functional recovery while chronic transplantation did not improve remyelination (Keirstead et al., 2005). In this study, contribution of invading SCs to remyelination was also assessed (Keirstead et al., 2005). The number of SC-remyelinated axons was significantly increased in injured animals and decreased to normal levels after transplantation of hESC derived OPC. In order to quantify the contribution of SCs and OPCs in remyelination, authors relied on the difference in the thickness of myelin to differentiate between central vs peripheral myelination. However, use of immunological labeling for SCs or ultrastructural criteria of SC myelination would be a more precise approach to determine the contribution of SCs in spinal cord remyelination after injury. In addition to SCs, endogenous OPCs and NPCs also contribute to remyelination which was not investigated in this study.

One important outcome of the Keirstead study was the inability of transplanted OPCs to remyelinate chronically demyelinated axons despite their survival and the capacity to differentiate into oligodendrocytes (Keirstead et al., 2005). This evidence suggests the inherited inhibitory nature of chronic lesions for axonal ensheathment and remyelination. Interestingly, our groups observed the same outcomes after transplantation of NPCs in chronic SCI (Karimi-Abdolrezaee et al., 2006). Our subsequent studies revealed that dense deposits of CSPGs in the chronic glial scar is a potent limiting factor to survival, integration, oligodendrocyte differentiation, and remyelination of both endogenous and transplanted NPCs (Karimi-Abdolrezaee et al., 2010, 2012; Dyck et al., 2015). As described in earlier sections, removal of CSPGs by ChABC allowed NPC-mediated remyelination of chronically demyelinated spinal cord axons. These studies suggest that successful application of OPCs or NPCs in chronic SCI requires multifaceted interventions to modulate the inhibitory milieu of the established glial scar. Although other factors may play a role in this inhibition, CSPGs seems to be a major obstacle (Dyck and Karimi-Abdolrezaee, 2015).

Efficacy of transplanted OPCs has also been investigated in chronic demyelinating conditions. Transplantation of mouse ESC derived OPCs into a radiation induced rat model of cervical spinal cord demyelination 4 months after radiation therapy, showed successful survival, and migration of these cells toward the lesion and their capacity for oligodendrocyte remyelination (Sun et al., 2013). Moreover, significant myelin and tissue preservation as well as improved forelimb locomotor function were observed in OPC transplanted animals (Sun et al., 2013). Transplantation of hESC derived OPCs transplanted into athymic nude rat model of radiation induced brain demyelination, promoted remyelination of the demyelinated brain and cerebellum and ameliorated cognitive and motor deficits in the injured animals, with no evidence of tumor formation following transplantation (Piao et al., 2015). Absence of neuronal differentiation and insignificant astrocyte differentiation in transplanted cells indicates a defining role for remyelination by oligodendrocytes in white matter repair and cognitive and motor improvement (Piao et al., 2015).

In addition to their role in replacing remyelinating oligodendrocytes, OPCs are known to enhance axo-neuronal growth and survival by producing growth factors such as brain derived neurotrophic factor (BDNF), IGF-1, glial derived growth factor (GDNF), neuregulins (NRGs), and neurotrophins (Du and Dreyfus, 2002; Dai, 2003; Wilkins et al., 2003) that potentially contributed to the functional improvement observed in these studies. Based on current evidence, transplanted OPCs or NPCs have additional benefits beyond their known role in cell replacement and myelination including improving the host environment with providing trophic support and their immunomodulatory effects.

### Olfactory Ensheathing Cells

Olfactory ensheathing cells (OECs) have been extensively examined for their potential for remyelination after SCI and demyelinating lesions (Ramon-Cueto and Nieto-Sampedro, 1994; Imaizumi et al., 2000; Kato et al., 2000; Akiyama et al., 2004; Radtke et al., 2004; Boyd et al., 2005; Sasaki et al., 2006, 2011; Lankford et al., 2008, 2014; Radtke, 2008; Tabakow et al., 2013). OECs are supporting cells that ensheath the axons of olfactory neurons. They have become a popular choice for cell transplantation due to their accessibility. Unlike oligodendrocytes or SCs, OECs do not normally myelinate axons in the nervous system (Sasaki et al., 2011). However, evidence suggests that following transplantation into the injured CNS, OECs demonstrate the ability to remyelinate axons and can promote functional recovery in animal models of SCI (Imaizumi et al., 2000; Kato et al., 2000; Akiyama et al., 2004; Radtke et al., 2004; Sasaki et al., 2006, 2011; Lankford et al., 2008; Radtke, 2008; Tabakow et al., 2013; Lankford et al., 2014).

Studies by Sasaki et al. (2011) showed evidence of remyelination by transplanted rat OECs in a rat model of X-EB induced demyelination in the thoracic spinal cord. Unlike chemical demyelination, X-EB models allow long lasting demyelination due to elimination of oligodendrocytes and astrocytes in the lesion area; therefore, excluding the potential contribution of endogenous myelinating cells. In this model, remyelination by transplanted OECs was investigated after acute and subacute OECs transplantation. Engrafted OECs were shown to integrate with the host tissue and remyelinated axons with predominant characteristic of P0 peripheral myelination (Sasaki et al., 2006). Immunoelectron microscopy at 2 and 3 weeks following transplantation confirmed OECs-derived remyelination of host demyelinated axons and reconstruction of the normal organization of the nodes of Ranvier (Sasaki et al., 2006). Functionality of OEC remyelination was confirmed by improved conduction velocity through electrophysiological recording of the spinal cords at 3 weeks after transplantation (Sasaki et al., 2006). While considerable myelination was observed following both acute and subacute lesions, the extent of myelination was considerably higher in acutely transplanted group (Sasaki et al., 2006).

Another study in nonhuman primate demonstrated the ability of OECs to remyelinate spinal cord axons following demyelination lesions (Radtke et al., 2004). In this study, porcine OECs were transplanted into chemically induced demyelinated adult female African green monkey spinal cord 3 days after the induction of lesion (Radtke et al., 2004). Evidence of remyelination was observed at 3-5 weeks after transplantation in 62.5% of the transplant recipients. Endogenously remyelinated axons were evident in the vicinity of OECs-myelinated axons in the lesion while no remyelination was observed in non-transplanted group at this time-point. However, this study was not able to clearly distinguish between OECs and SCs derived remyelination (Radtke et al., 2004).

Since SCs and OECs demonstrate similar characteristics, their behavior and response have been compared in demyelinated spinal cord in co-transplantation studies (Lankford et al., 2008, 2014). Interestingly, these studies suggest distinct migratory and proliferative properties for OECs compared to SCs. Lankford et al. (2008) injected a mixture of SCs and OECs at 1 week after X irradiation into the rat spinal cord. Only transplanted OECs migrated through white and gray matter of the irradiated spinal cord. Four weeks after transplantation, a new EB demyelination was induced in the spinal cord. Tissue analysis revealed that “pre-loaded” OECs but not SCs were able to migrate toward the new EB lesion and populate the demyelinated site. Subsequent studies by the same induced demyelination using X irradiation in the juvenile rats hippocampus confirmed a better migratory ability for OECs in populating the lesion compared to SCs similar to the pattern observed in the adult host (Lankford et al., 2008, 2014).

While extensive evidence suggests a direct role for OECs in axonal ensheathing and remyelinating, there are also some reports that have questioned their myelinating capability in SCI (Takami et al., 2002; Boyd et al., 2004). Boyd et al. (2004), using Lacz expressing OECs, were able to track transplanted OECs in the spinal cord of injured rats. While labeled OECs were evident in the lesion at 3 weeks post-transplantation, electron micrographs of the injured spinal cord showed no direct evidence of axonal ensheathment and myelination by LacZ labeled OECs (Boyd et al., 2004). Transplanted LacZ labeled OECs mainly enveloped a group of axons myelinated by SCs. The authors concluded that OECs support SCI repair by other mechanisms such as providing permissive substrate for axon growth and SC remyelination (Boyd et al., 2004). In contrast to these observations, another study using superparamagnetic iron oxide labeling demonstrated the ability of both transplanted SCs and OECs for remyelination in X-EB spinal cord demyelination (Dunning et al., 2004). Subsequently, Dombrowski et al. (2006) transplanted GFP expressing OECs into the injured sciatic nerve and using immunoelectron microscopy showed that transplanted GFP+ OECs formed peripheral type myelin around axons. However, one question that remained undetermined in these studies was the degree of SC contamination in their OEC culture. Degree of SCs contamination in OEC cultures is an important consideration that may underlie different outcomes seen in these studies.

Regardless of their direct role in axonal remyelination, OECS are shown to recondition injury environment by producing a host of trophic factors including NGF, BDNF, and CNTF that can support endogenous repair (Ramon-Cueto and Avila, 1998; Chuah and West, 2002; Martin et al., 2002; Au and Roskams, 2003). Studies have shown that transplanted OECs enhance functional recovery through enhanced angiogenesis and immunomodulatory effects at acute and subacute stages post-transplantation (López-Vales et al., 2005). López-Vales et al. (2005) demonstrated that transplantation of OECs results in upregulation of Cox-2 and vascular endothelial growth factor (VEGF) following SCI.

Collectively, while application of OECs has shown promising results in experimental models of SCI and demyelination, the ability of OECs for CNS remyelination is still a matter of debate due to their similarities with SCs and potential contamination of OEC cultures with SCs. Further *in vitro* and *in vivo* studies using specific markers of OECs and SCs is required to specifically distinguish these two cell populations and confirm the myelinating capacity of OECs.

### Induced Pluripotent Stem Cells

While cell translation is a promising approach for enhancing remyelination following injury, graft rejection due to the host immune reaction poses a major challenge to the success of cell-based therapies (Nakamura and Okano, 2013). Successful transplantation in animal models has required immunosuppression, wherein chance of the graft rejection had been minimized using immunosuppressive therapy or genetically immune-deficient models (Cummings et al., 2005; Karimi-Abdolrezaee et al., 2006, 2010). Importantly, immune reaction is a hallmark of pathologic CNS that plays essential roles in tissue injury and repair (Eyo and Dailey, 2013; Cherry et al., 2014). Hence, use of autologous sources of stem cells seems to be a logical solution to warrant the long-term survival of transplanted cells without compromising the host CNS immune response. Moreover, autologous transplantation preclude ethical and technical limitations in using embryonic stem cells or fetal derived tissues that has been a major hurdle in translating this promising repair strategy into clinical arenas (Lo and Parham, 2009; Zarzeczny and Caulfield, 2009; Nakamura and Okano, 2013; King and Perrin, 2014).

Generation of induced pluripotent stem cells (iPSCs) using autologous somatic cells has opened new avenues for developing clinically feasible cell transplantation approaches (Saito et al., 2005; Takahashi and Yamanaka, 2006; Yamanaka and Takahashi, 2006; Qi and Pei, 2007; Yamanaka, 2008; Nagy, 2009; Zhou et al., 2010; Liu et al., 2012; Nguyen et al., 2014). Since the introduction of iPSCs technology in stem cell research, several studies have explored the therapeutic potential of transplanting iPSCs-derived NPCs or OPCs in SCI for inducing remyelination as well as the safety of this approach with regards to tumorigenesis (Miura et al., 2009; Tsuji et al., 2010; Nori et al., 2011; Kobayashi et al., 2012; Wang et al., 2013). Tsuji et al. (2010) transplanted allogenic NPCs generated from mouse embryonic fibroblast (MEF)-derived iPSCs into a mouse model of contusive SCI at 9 days post injury. The safety of this approach was previously confirmed by observing no teratoma formation after 24 weeks of transplantation into NOD/SCID mouse brain (Miura et al., 2009). Six weeks following transplantation, MEF-iPSC derived NPCs showed multilineage capacity and differentiated into oligodendrocytes, astrocytes, and neurons (Tsuji et al., 2010). Functional analysis suggested improved neurological recovery in the transplanted animals, which was accompanied by enhanced remyelination as well as regeneration of the raphe-spinal fibers in transplanted animals (Tsuji et al., 2010). Similar observations have been made using a variety of adult tissue derived iPSCs (Nakamura and Okano, 2013). Transplantation of selected non-tumorigenic clone of adult tail tip fibroblast derived iPSCs into a mouse model of contusive SCI resulted in significant functional recovery (Tsuji et al., 2010; Nakamura and Okano, 2013). In mouse (Nori et al., 2011) and primate (Kobayashi et al., 2012) models of SCI, transplanted NPCs derived from human adult facial skin derived iPSCs were able to differentiate into neurons, oligodendrocytes, and astrocytes which resulted in enhanced functional recovery accompanied by axonal growth (Nori et al., 2011; Kobayashi et al., 2012; Nakamura and Okano, 2013).

Remyelination has been considered as a mechanism underlying the functional improvement observed in iPSCs transplantation strategies (Wang et al., 2013). A recent study by Wang et al. (2013) demonstrated that transplanted OPCs generated from autologous skin derived human iPSCs of keratinocyte and fibroblast origin, were able to differentiate into mature myelinating oligodendrocytes and myelinate human fetal cortical neurons *in vitro* (Wang et al., 2013). In immune-deficient *Shiverer* mice, human iPSC-derived OPCs integrated with the host neonatal brain tissue after transplantation, differentiated to MBP expressing oligodendrocytes, remyelinated the dysmyelinated *Shiverer* axons, and constructed the nodes of Ranvier (Wang et al., 2013). No tumorigenicity was observed in this study and transplanted *Shiverer* mice exhibited increased lifespan (Wang et al., 2013).

Taken together, emerging evidence shows the potential and feasibility of transplanting iPSCs derived NPCs and OPCs for promoting oligodendrocyte replacement and remyelination in demyelinating conditions such as MS and traumatic CNS injury. While promising, the risk of tumorigenicity and proper cell differentiation of this therapy have yet remained to be carefully investigated. In **Table 1**, we have listed cell therapies that have been developed to promote oligodendrocytes replacement and remyelination after SCI and MS conditions.

## Conclusion

The myelin sheath is an essential component in the CNS and PNS that ensures rapid signal transduction in axons through the nervous system. Myelinated fibers show a highly specialized and organized structure at the node of Ranvier that is vital to their proper functioning. Damage to oligodendrocytes due to trauma or disease results in demyelination that causes aberrant localization and expression of ion channels associated with axonal dysfunction. Although considerable remyelination occurs spontaneously by endogenous CNS progenitor cells, the quality and extend of myelination is not optimal to restore structural and physiological properties of injured axons. Emerging research evidence has uncovered several mechanisms by which oligodendrocyte differentiation and remyelination are regulated within the microenvironment of injury. Inhibitory role of activated glial cells and insufficient expression of promoting factors are major limiting factors that impede remyelination. Over the past years, extensive research efforts have been made to therapeutically promote remyelination following CNS trauma or demyelinating disease. Outcomes of the recent pharmacological and cell-based therapies indicate the impact of remyelination as a mechanism for improving function after injury. Further investigations are needed to develop effective and feasible repair strategies with potential for clinical translation.

## Conflict of Interest Statement

The authors declare that the research was conducted in the absence of any commercial or financial relationships that could be construed as a potential conflict of interest.
